# Fibromyalgia: A Multifactorial Pain Disorder

**DOI:** 10.3390/ijms27114813

**Published:** 2026-05-27

**Authors:** Tomohiko Aoe

**Affiliations:** 1 Pain Center, Chiba Medical Center, Teikyo University, Ichihara 299-0111, Japan; aoe.tomohiko.id@teikyo-u.ac.jp; 2 Pain Clinic, Sanmu Medical Center, Sanmu 289-1326, Japan

**Keywords:** fibromyalgia, nociplastic pain, chronic pain, attention network, post-viral fatigue syndrome

## Abstract

Fibromyalgia represents a complex chronic pain condition in which patients experience widespread pain accompanied by fatigue and a broad range of physical and psychological symptoms. Diagnosis relies on symptom-based diagnostic criteria that lack objectivity, making it difficult to properly diagnose and treat. The etiology of fibromyalgia is thought to be multifactorial, including neurobiological, psychological, environmental, and genetic factors. In addition to pain, many patients experience central nervous system-related symptoms, such as fatigue, sleep disturbances, cognitive impairment, anxiety, and depression. Fibromyalgia has recently been recognized as a nociplastic pain disorder. Alterations in sensory processing and pain thresholds in the central nervous system may result in the perception of widespread pain throughout the body. In a recent study, we divided fibromyalgia patients who reported widespread pain into two subgroups based on their pain threshold: a low pain threshold group and a normal pain threshold group. We comparably analyzed these subgroups by using resting-state functional magnetic resonance imaging. This hypothesis article provides an overview of the pathogenesis of fibromyalgia and discusses these two subgroups of nociplastic pain. Recognizing the various pathophysiologies of fibromyalgia will allow us to better understand patients’ conditions and select appropriate treatment options for each patient.

## 1. Introduction

Fibromyalgia (FM) remains one of the most challenging chronic pain conditions in clinical practice, owing to its heterogeneous symptom profile and the absence of objective diagnostic biomarkers. Clinically, FM manifests as diffuse pain affecting both axial and appendicular regions, often co-occurring with fatigue, sleep disruption, cognitive complaints, and affective symptoms such as anxiety and depression [1]. These diverse manifestations often lead to diagnostic uncertainty and delayed initiation of appropriate treatment.

Epidemiological studies indicate that FM affects a substantial proportion of the population, with reported prevalence rates of approximately 5.4% in the United Kingdom [2], 2.1% in Japan [3], and a pooled global prevalence of 1.78% based on a large-scale meta-analysis [4]. Despite its relatively high prevalence, FM is frequently underdiagnosed or misclassified, reflecting fundamental limitations in current diagnostic frameworks.

The most widely used diagnostic criteria for FM are those proposed by the American College of Rheumatology (ACR). Earlier ACR frameworks emphasized examiner-based tender point assessment [5], whereas later revisions have increasingly prioritized patient-reported pain distribution and associated symptoms such as fatigue and cognitive dysfunction [1,6,7]. Although these revisions improved clinical feasibility, they also introduced greater reliance on subjective symptom reporting, raising concerns regarding diagnostic objectivity and inter-clinician variability. This reliance on subjective criteria can contribute to diagnostic ambiguity for both patients and healthcare professionals, particularly in clinical settings where FM overlaps with other chronic pain, autoimmune, or functional disorders. This hypothesis paper argues that it is preferable to recognize FM not as a single disease entity, but as a heterogeneous syndrome encompassing multiple pathophysiological mechanisms.

## 2. Pathophysiological Classification of Pain and Fibromyalgia

Based on pathophysiology, pain is classified as nociceptive pain, neuropathic pain, or nociplastic pain [8,9]. Furthermore, a category called “mixed pain,” which involves a combination of these pain types, has been proposed [10]. Considering the various etiologies of FM is important for developing treatment strategies tailored to individual patients. The pain experienced by patients with FM is primarily classified as nociplastic pain, characterized by altered pain processing in the central nervous system (CNS) without clear evidence of tissue damage or inflammation [11]. However, FM may be a multifactorial pain syndrome, with various contributions from nociceptive, neuropathic, and nociplastic pain.

### 2.1. Fibromyalgia and Nociceptive Pain

Nociceptive pain resulting from actual or potential tissue damage may contribute to the clinical manifestations of many patients with FM [9]. While central sensitization is the mainstay of FM pathophysiology, peripheral pain-generating factors, such as neurogenic inflammation, nociceptor sensitization, and altered muscle metabolism, may function as ongoing nociceptive input and perpetuate central sensitization [12].

#### 2.1.1. Autoimmune Mechanisms

FM has been associated with autoimmune diseases such as rheumatoid arthritis [13] and vaccinations [14]. Recent evidence suggests that some patients with FM have autoantibodies targeting gangliosides, transient receptor potential vanilloid 1 (TRPV1), serotonin 5-HT1A receptors, GABA receptors or fibroblast growth factor 3 [15,16]. Transplantation of IgG from FM patients into mice has been shown to induce hyperalgesia in the mice, and immunohistochemical analysis showed that the IgG was deposited in the dorsal root ganglia, suggesting that an IgG-mediated mechanism may contribute to the development of symptoms in some patients [17]. Cohort studies from Sweden and Canada have reported that patients with FM who have elevated levels of anti-satellite glial IgG antibodies exhibit more severe symptoms [18]. Another study suggests that levels of autoantibodies against various nerve- and tissue-specific antigens correlate with the severity of fatigue, bodily pain, depression, anxiety, and physical and mental health-related quality of life [15].

#### 2.1.2. Altered Muscle Metabolism

Morphological and metabolic changes have been observed in the muscles of patients with FM, which, while not specific to FM, suggest the involvement of peripheral factors in maintaining pain intensity [19]. Studies using phosphorus-31 magnetic resonance spectroscopy have demonstrated mitochondrial dysfunction and reduced oxygen utilization in the skeletal muscle of these patients [20]. Studies of peripheral blood mononuclear cells have shown that mitochondrial function is impaired in patients with FM, which correlates with symptom severity [21]. These changes lead to the accumulation of lactate and pyruvate, thereby enhancing peripheral nociceptive input.

#### 2.1.3. Neurogenic Inflammation

Patients with FM have elevated concentrations of neuropeptides, such as substance P [22] and calcitonin gene-related peptide (CGRP) [23], in their cerebrospinal fluid and serum. These mediators promote vasodilation, plasma extravasation, and mast cell activation, contributing to local hyperalgesia and tenderness [24].

#### 2.1.4. Sensitization of Peripheral Pain Receptors

In patients with FM, reduced muscle capillary density and blood perfusion have been observed, suggesting a reduced oxygen supply, especially during exercise. Local hypoxia may amplify peripheral pain signals [25]. Furthermore, microneurography and skin biopsy findings have revealed hyperexcitability of C nociceptors and reduced intraepidermal nerve fiber density in some patients with FM [19]. These data support the hypothesis that peripheral ischemia and small fiber pathology may interact to perpetuate peripheral sensitization and chronic pain [26].

### 2.2. Fibromyalgia and Neuropathic Pain

Neuropathic pain results from lesions or diseases in the somatosensory nervous system and involves abnormalities in pain processing [9]. Many patients with FM present with neuropathic symptoms, including burning pain, paresthesia, allodynia, and hyperalgesia. FM often encompasses features of both nociplastic and neuropathic pain [27].

#### 2.2.1. Genetic Factors

Genetic studies of FM suggest that abnormalities in the nervous system may sometimes be involved in its development. FM tends to cluster in families, suggesting a genetic component [28]. First-degree relatives of patients with FM have been shown to have up to an eightfold increased risk [29]. Genetic association studies have identified polymorphisms in the serotonin transporter gene (*5-HTTLPR*) [30], the catechol-O-methyltransferase gene (*COMT*) [31], and the dopamine receptor D4 gene (*DRD4*) [32], all of which are involved in pain and mood regulation. However, there is no evidence that FM is caused by a single gene mutation [33,34].

Epigenetic mechanisms, such as DNA methylation, may also be involved in FM [35]. Compared with healthy controls, patients with FM have been reported to exhibit hypo-methylation in pathways such as signal transduction, calcium signaling, the MAPK pathway, immune response, and cellular stress response/oxidative stress response [36]. These modifications may be caused by chronic stress or trauma and may alter gene expression in pain-related pathways. An Epigenome-Wide Association Study examining differences in heat pain tolerance between healthy monozygotic twins reported that higher methylation status of the transient receptor potential ankyrin 1 (*TRPA1*) promoter region was associated with higher pain sensitivity [37].

#### 2.2.2. Small Fiber Neuropathy

Small fiber neuropathy involves damage to unmyelinated C fibers and thinly myelinated Aδ fibers. These fibers regulate pain, temperature, and autonomic function. Small fiber neuropathy has been detected in up to 40–60% of patients with FM and is supported by reduced intraepidermal nerve fiber density in skin biopsies and abnormal findings on corneal confocal microscopy [38]. A recent meta-analysis reported that approximately half of patients with FM had small fiber dysfunction [39,40]. Two distinct pathologies may exist: impaired small-fiber nerves and reduced innervation [41]. Skin biopsies have demonstrated a significant reduction in unmyelinated C fibers in patients with FM [42]. Small fiber dysfunction can lead to neuropathic pain symptoms, such as burning and tingling, as well as autonomic dysfunction (e.g., orthostatic intolerance, sweating abnormalities, and gastrointestinal disorders) [39]. However, small fiber neuropathy is likely to characterize a specific subset of FM, rather than being a universal feature.

#### 2.2.3. Peripheral Nerve Regeneration Failure and Neurotrophic Factor Imbalance

Peripheral nerve injury typically triggers blastema formation and synaptic remodeling. Elevated nerve growth factor (NGF) levels have been reported in FM and other chronic pain conditions [43,44]. NGF, an important neurotrophic factor, binds to the tropomyosin receptor kinase A (TrkA) receptor and promotes pain transmission by increasing the expression of sodium channels (Nav1.7, Nav1.8) and TRPV1 [45]. Elevated NGF levels in patients with FM may contribute to peripheral and central sensitization, amplifying pain signals. Thus, anti-NGF therapy has shown promise for pain relief in chronic pain conditions [46]. Molecular and neurophysiological data collectively support the notion that impaired nerve repair and excessive NGF-TrkA signaling may contribute to persistent pain in susceptible patients; however, direct histopathological evidence in FM remains limited [47].

#### 2.2.4. Endogenous Opioid Dysregulation

Opioids are not recommended for use in patients with chronic pain, such as FM [48,49]. Opioid use can lead to tolerance development and, relatively early, opioid-induced hyperalgesia [50]. In opioid-induced hyperalgesia, the pain threshold is lowered, and clinically, the symptoms resemble those of FM. When tolerance develops at μ-opioid receptors expressed on inhibitory interneurons of the descending pain inhibitory system, such as the periaqueductal gray (PAG), systemic hyperalgesia occurs due to dysfunction of the descending pain inhibitory system [51]. In fact, resting state functional magnetic resonance imaging (MRI) studies have shown dysfunction of the descending pain inhibitory system in FM patients [52].

#### 2.2.5. Neuroinflammation

Activation of microglial cells and astrocytes in the CNS appears to be a hallmark of low-grade neuroinflammation in FM. Positron emission tomography (PET) imaging in FM patients showed increased translocator protein (TSPO) binding in cortical regions, including the frontal and parietal lobes, suggesting widespread activation of microglial cells [53]. Neuroinflammation has been shown to be heterogeneously distributed in patients with FM. Increased inflammation was observed in the primary motor cortex, primary somatosensory cortex, right postcentral gyrus, left superior parietal gyrus, superior frontal gyri, left precuneus, and left medial frontal gyrus, while decreased inflammation was observed in the medulla, left superior temporal gyrus, and left amygdala [54]. MRI-based diffusion kurtosis imaging (DKI), which quantifies microstructural alterations in neuroinflammation, revealed decreased kurtosis parameters in the dorsolateral prefrontal and orbitofrontal cortices, as well as a correlation between amygdala changes and pain severity [55]. Lower DKI parameters are thought to reflect chronic neuroinflammation-related tissue damage, including axonal damage, impaired myelination, and glial-mediated structural disorganization [55]. These findings indicate that FM is accompanied by persistent microstructural brain changes.

Beyond imaging, biochemical evidence supports neuroinflammatory activity in FM. Activated microglia and astrocytes release cytokines (e.g., IL-6, IL-8, IL-10, and TNF-α) that sensitize neurons and promote central pain amplification [56,57]. Analysis using a multiplex protein panel revealed elevated levels of IL-8 in cerebrospinal fluid and plasma from patients with FM relative to healthy controls, as well as elevated levels of the chemokine CX3CL1 (also known as fractalkine) [58]. Cathepsin S inhibitors may block cathepsin S-mediated CX3CL1 cleavage, suppressing persistent microglial activation and reducing hyperalgesia [59].

Meanwhile, elevated serum Aβ_1–42_ and tau levels have been reported in FM, suggesting that glial activation and neuroinflammation may contribute to subtle neurodegenerative processes [55]. Furthermore, oxidative and mitochondrial dysfunction have been implicated as upstream triggers of neuroinflammation, suggesting that disorders of cellular energy metabolism may be related to central pain amplification [60]. Neuroinflammation in FM not only affects pain, but also psychological symptoms. PET studies have revealed that neuroinflammation in the left medial frontal, left superior frontal, and left amygdala is correlated with psychological stress dysregulation [54]. However, the causal direction between neuroinflammation and FM symptoms remains controversial, as it is still unclear whether glial activation represents a primary driver of symptom development or a secondary consequence of prolonged nociceptive and emotional stress.

#### 2.2.6. Post-Viral Fatigue Syndrome and Fibromyalgia

FM has been reported to develop following viral infections [61]. In recent years, one of the most common causes of neuroinflammation associated with FM symptoms is thought to be post-viral fatigue syndromes such as SARS-CoV-2 infection, long COVID or Epstein–Barr virus infection as chronic fatigue syndrome [62,63,64]. In addition to sequelae of acute organ damage, such as pulmonary fibrosis, cardiac dysfunction, and renal dysfunction, long COVID symptoms include fatigue, cognitive impairment known as brain fog, anxiety, sleep disturbances, headaches, and orthostatic hypotension, which have been observed even in patients with mild or asymptomatic cases who did not require hospitalization during the acute phase [62]. These symptoms closely resemble those of a condition called chronic fatigue syndrome/myalgic encephalomyelitis [65]. Chronic fatigue syndrome was known as a disorder primarily characterized by fatigue, but there are no objective diagnostic criteria. Diagnosis is based on clinical symptoms [66]. Chronic fatigue syndrome is classified as 8E49 Postviral fatigue syndrome in the International Classification of Diseases 11th Revision (ICD-11). Some FM patients meet the diagnostic criteria for both chronic fatigue syndrome and FM [67]. Long COVID complications sometimes involve widespread pain, which may also meet the diagnostic criteria for FM [65].

In long COVID, neuroinflammation is suspected to be caused by several factors, such as chronic persistent infection with SARS-CoV-2 [65], the activation of autoantibodies and immune cells that react to the nervous system due to the immune response to SARS-CoV-2 [68], and the reactivation of endogenous latent viruses such as human herpesvirus 6, human herpesvirus 7 and Epstein–Barr virus [69]. Some FM patients may share similar pathological conditions with those of long COVID and chronic fatigue syndrome. Neuroinflammation and glial activation caused by viral infection or immune responses can lead to cytokine release and oxidative stress [70], potentially causing endoplasmic reticulum stress [71] and mitochondrial dysfunction [60] in neurons. When the brainstem is damaged by neuroinflammation, dysfunction of the descending pain inhibitory system, autonomic nuclei, reticular formation, and other systems can lead to a variety of symptoms, including hyperalgesia, fatigue, orthostatic hypotension, and brain fog [72]. If other areas of the central or peripheral nervous system are damaged, various symptoms may appear as focal symptoms in the affected area.

### 2.3. Fibromyalgia and Nociplastic Pain

FM is increasingly recognized as a prototypical nociplastic pain disorder [11]. Nociplastic pain is characterized by abnormalities in pain processing without actual tissue damage or overt somatosensory involvement [9]. Unlike nociceptive or neuropathic pain, nociplastic pain is thought to arise from dysfunctional pain modulation in the CNS. The following mechanisms are thought to be involved.

#### 2.3.1. Central Sensitization

Patients with FM commonly demonstrate hyperexcitability of CNS neurons involved in pain processing. Functional MRI studies have demonstrated increased connectivity and activity in several brain regions, such as the insular cortex [73], amygdala [74], and somatosensory cortex [75]. FM often manifests as widespread pain hypersensitivity [76]. Patients with FM exhibit elevated concentrations of glutamate, a major cortical excitatory neurotransmitter, in the right posterior insula [77] and substance P in the cerebrospinal fluid [22], potentially indicating enhanced pain signaling. FM patients also show impaired conditioned pain modulation, suggesting dysfunction of the descending pain inhibitory system [78]. Resting state functional MRI has demonstrated dysfunction of the descending pain inhibitory system in patients with FM who experience widespread pain [52,79]. This dysfunction is mediated in part by decreased monoamine neurotransmission. These patients have decreased cerebrospinal fluid concentrations of serotonin, noradrenaline, and dopamine, which are involved in endogenous pain inhibition [80]. These neurochemical abnormalities may not only modulate pain perception but also contribute to the mood and sleep disturbances frequently observed in FM [81]. Serotonin and noradrenaline reuptake inhibitors (SNRIs), such as duloxetine [82] and milnacipran, have demonstrated efficacy in enhancing endogenous inhibition and reducing pain intensity [81,83].

#### 2.3.2. Psychopathological Factors

Psychological and socio-emotional factors may also play a role [84]. A history of adverse childhood experiences, such as emotional abuse and neglect, has been reported to be associated with an increased risk of chronic pain, including juvenile FM [85,86]. Comorbid emotional disorders such as anxiety, depression, and somatization are common in patients with FM, exacerbating the severity of symptoms [87]. A correlation between certain affective spectrum disorders and the severity of FM symptoms has been reported [88]. Neuroendocrine dysregulation, such as hypothalamic–pituitary–adrenal (HPA) axis dysfunction and flattened cortisol response, may exacerbate fatigue and stress reactivity [89].

#### 2.3.3. Interactions Between Nociplastic Pain and Nociceptive or Neuropathic Pain

Continuous nociceptive input from peripheral tissues, such as soft tissues and myofascia, can induce and maintain central sensitization, potentially leading to a transition from nociceptive pain to nociplastic pain. Blocking nociceptive input with local anesthesia has been shown to temporarily relieve pain in FM [90]. Clinically, FM often overlaps with chronic nociceptive pain disorders such as osteoarthritis and rheumatoid arthritis [91,92]. Furthermore, FM often encompasses features of a mixed-pain condition with both nociplastic and neuropathic pain [27]. Even if commonly used clinical blood tests and imaging diagnostics indicate “no actual tissue damage or obvious lesions in the somatosensory system,” molecular pathological testing [58], PET [53,54] or MRI imaging [55] may reveal additional findings. It is difficult to regard neuropathic pain and nociplastic pain as mutually exclusive entities, especially in everyday clinical practice.

## 3. Subclasses of Nociplastic Pain in Fibromyalgia

**Figure 1:**
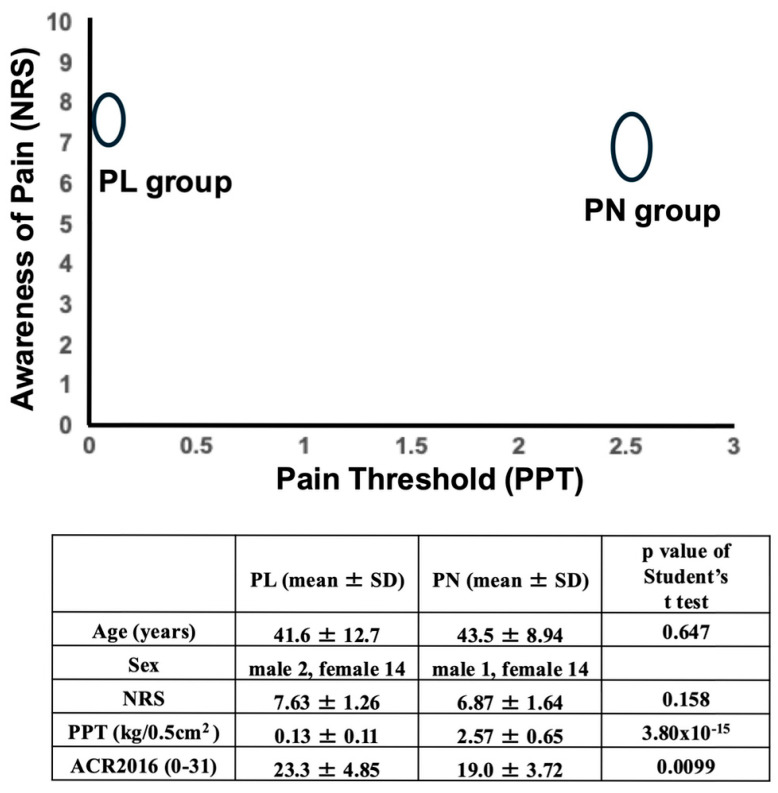
A significant difference between the two FM subgroups for pain threshold. Based on pressure pain threshold (PPT) values, 16 patients were assigned to the PL group (low pain threshold) and 15 patients were assigned to the PN group (normal pain threshold). The patients in both groups complained of similar widespread pain and were diagnosed with FM based on the American College of Rheumatology (ACR) 2016 criteria [6]. There was a marked difference in their thresholds to pain. The numbers represent the mean ± SD. Values in the PL and PN groups were analyzed by Student’s *t* test [76]. The pain threshold in the PN group was equivalent to that in subjects with no pain, as determined in a previous study (25 females, age 42.6 years (±7.3), pressure pain threshold 2.44 (±0.75), Student’s *t* test; *p* = 0.572 vs. PN group) [93]. This figure was created based on the paper by Aoe et al., Scientific Reports, 2024 [76] under the terms of the Creative Commons Attribution 4.0 International License. Changes were made to the original figure. PL: low pain threshold; PN: normal pain threshold; SD: standard deviation; NRS: numerical rating scale; PPT: pressure pain threshold; ACR: American College of Rheumatology.

Each patient with FM may experience nociplastic pain owing to a combination of various etiologies that alter pain perception. In treating patients with FM, we noticed that some patients exhibited significant hyperalgesia, while others showed pain thresholds similar to those of healthy individuals. In a recent study, we considered the possibility that FM patients could be divided into subgroups based on their pain threshold and examined the characteristics of these two subgroups [76] (Figure 1). All patients were diagnosed according to the 2016 ACR criteria based on subjective pain perception and other subjective symptoms [6]. In this diagnostic criterion, patients report widespread pain, but hyperalgesia is not necessarily present.

1. Low Pain Threshold (PL) Group: Patients in this subgroup have significantly reduced pressure pain thresholds (PPTs) and increased nociceptive sensitivity.
2. Normal Pain Threshold (PN) Group: Despite PPTs within the normal range, these patients report pain as severe as the PL group.

In this study, resting state functional MRI-based functional connectivity analysis was employed to characterize network-level differences between FM subgroups. Previous imaging studies have revealed that peripheral pain stimuli activate multiple brain regions, including somatosensory, insular, and cingulate areas, as well as frontal and parietal areas. These regions are collectively known as the pain matrix [94]. When comparing pain-free healthy subjects with pain-experiencing patients, it is unclear whether specific brain regions are activated as a result of pain stimuli or whether activation of these regions contributes to the perception of pain in these patients. Therefore, we investigated differences in functional connectivity between these two patient subgroups, which had similar levels of pain. Key findings revealing significant differences between the two patient groups include the following [76].

1. Functional connectivity between the secondary somatosensory cortex (S2) and the dorsal attention network (DAN) was significantly greater in the normal pain threshold group, possibly suggesting enhanced top-down, goal-driven pain perception via working memory.
2. In the low pain threshold group, functional connectivity between the thalamus and insular cortex was significantly greater, suggesting enhanced bottom-up pain signaling via the spinothalamic tract. There was also increased connectivity between the ventral attention network (VAN) and the DAN.
3. In the low pain threshold group, functional connectivity between the insular cortex and PAG was significantly smaller, suggesting impairment of the descending pain inhibitory system underlying hyperalgesia.

This functional MRI study was performed at rest, in the supine position, and without painful stimulation. Standard imaging studies did not reveal clear lesions that would explain the patients’ complaints of pain. In the PN group, significantly greater functional connectivity was observed between S2 and DAN (the left parietal opercular cortex (S2) and the intraparietal sulcus of the left dorsal attentional network, PL: 0.113 (0.05–0.18); PN: 0.448 (0.32–0.58); *p* = 7.38 × 10^−5^; effect size: 1.66, p-FDR = 0.012) [76]. The PL group patients experienced hyperalgesia even in the supine position, particularly in areas under gravity. If painful stimuli were applied, even only slight sensory stimuli that healthy individuals would not perceive as painful, patients in the PL group would report more intense pain. Under these circumstances, significant changes would be expected to be detected by functional MRI in the PL patients. The pain threshold of the PN group was no different from that of healthy individuals. Therefore, changes in response to painful stimuli would be expected to be similar to those in healthy individuals.

**Figure 2:**
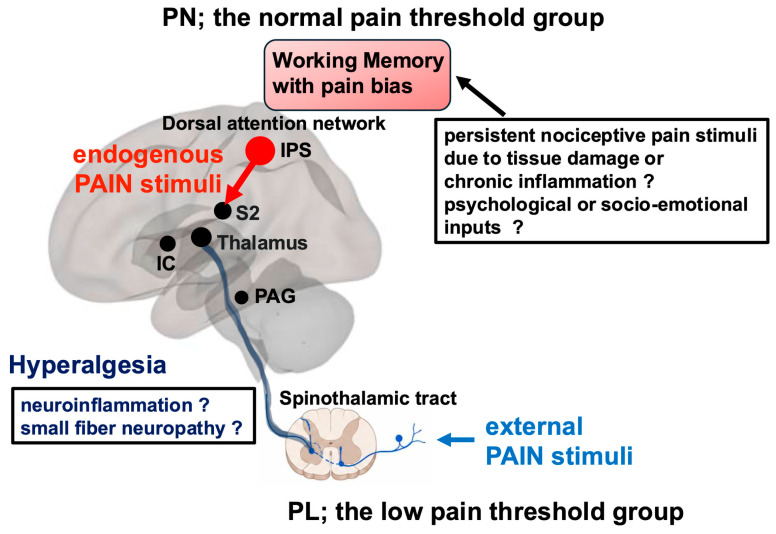
A hypothetical model of nociplastic pain subclasses in fibromyalgia. The normal pain threshold group (PN) may experience endogenous pain due to stimulation of the S2 area by working memory formed in the dorsal attention network (DAN), whereas the low pain threshold group (PL) may perceive ongoing external sensory stimuli through the spinothalamic tract as pain due to hyperalgesia caused by impairment of the descending pain inhibitory system or by other mechanisms such as small fiber neuropathy. This figure was created based on the paper by Aoe et al., Scientific Reports, 2024 [76] under the terms of the Creative Commons Attribution 4.0 International License. Changes were made to the original figure. IPS DAN: intraparietal sulcus in the dorsal attention network; IC: insular cortex; S2: the secondary somatosensory area, PAG: periaqueductal gray.

Functional connectivity suggests functional connections between arbitrary neural regions, but it does not necessarily prove the direction of information transmission or a causal relationship with neural activity or cognitive function. This study involved a small number of patients and requires confirmation in a larger-scale study, but it raised the following hypotheses. Although both of these subgroups present with nociplastic pain, their underlying pathophysiology appears to differ. The parietal operculum, also known as S2 [95], and the posterior insular cortex are thought to be core areas for pain sensation [96]. It has been hypothesized that the group with a normal pain threshold (PN) may perceive endogenous pain through stimulation of the S2 region via working memory formed in the DAN. On the other hand, the group with a low pain threshold (PL) may have altered pain perception in the descending pain inhibitory system or other regions associated with pain perception, leading to hyperalgesia in response to external sensory stimuli [76] (Figure 2).

## 4. Discussion

### 4.1. Attention Networks and Fibromyalgia

Attention is a core cognitive function that enables goal-directed behavior. Two major large-scale brain networks support attentional control [97]:

(1) DAN: mediates goal-directed, top-down attention.
(2) VAN: mediates stimulus-driven, bottom-up attention.

The DAN includes the dorsal parietal cortex, particularly the intraparietal sulcus (IPS) and superior parietal lobule, and dorsal frontal cortex along the precentral sulcus [98]. The DAN is responsible for maintaining attention to task-relevant goals and enabling voluntary control of sensory processing [98]. The VAN includes the temporoparietal junction cortex, the right middle frontal gyrus, inferior frontal gyrus, frontal operculum, and anterior insula [98]. Unlike the DAN, the VAN is activated upon detection of unexpected, behaviorally relevant stimuli from the external environment, triggering attentional switching [98]. These two systems dynamically interact, with signals via the VAN redirecting information from the DAN, allowing better responses to the external environment [98]. Pain is thought to be one type of sensory information processed by these two attention networks [99,100]. Painful stimuli, such as the cold pressor test, activate the VAN [101]. Some patients with FM who exhibit hyperalgesia may experience intense pain in response to external stimuli and are therefore likely to show enhanced VAN responses. Increased functional connectivity from the right middle frontal gyrus of the VAN to the DAN was observed in patients with a lowered pain threshold, suggesting the enhanced sensory information from external stimuli [76].

Patients in the low pain threshold group may appear to have nociplastic pain while also possessing elements of neuropathic pain, such as hyperalgesia and allodynia. Although this study did not include detailed neurophysiological examinations, neuropathic pain assessments, or biopsies of small fiber neuropathy, the following hypotheses could be considered.

In the normal pain threshold group, persistent nociceptive pain stimuli, such as tissue injury or chronic inflammation, and psychological or socio-emotional inputs might have entered the VAN via physiological neurotransmission pathways. Subsequently, the VAN-DAN interactions might form working memory with pain-related attentional bias in the DAN. These patients may perceive endogenous pain by stimulation of the S2 region via working memory formed in the DAN (Figure 2). If nociplastic pain is defined as “Pain that arises from altered nociception despite no clear evidence of actual or threatened tissue damage causing the activation of peripheral nociceptors or evidence for disease or lesion of the somatosensory system causing the pain” [8], the normal pain threshold group is considered a more typical example of nociplastic pain. The low pain threshold group may have altered pain perception due to some form of neural dysfunction in the descending pain inhibitory system or other areas related to pain perception including small fiber neuropathy, resulting in hyperalgesia in response to ongoing external sensory stimuli [76] (Figure 2). In such cases, neurological disorders that sometimes cannot be detected by clinical routine blood tests or imaging may be involved.

### 4.2. Pathophysiology-Based Personalized Medicine for Fibromyalgia

FM may not be a disease caused by a single, uniform etiology, but rather a syndrome that can be caused by multiple etiologies. Several etiologies may overlap, with the combination of etiologies potentially differing from patient to patient. Therefore, while wide-spread pain is common, there are various differences in other symptoms. Patients with FM experience chronic pain and a variety of symptoms, necessitating more personalized treatment strategies tailored to their individual pathophysiology.

Pressure pain thresholds (PPTs) can be easily measured in an outpatient setting using a pressure meter [76]. This may help determine whether a patient falls into the low pain threshold group, the normal pain threshold group, or somewhere in between. While biomarkers specific to FM are difficult to identify, measuring cytokines such as IL-8, IL-10 and TNFα in peripheral blood may help in inferring the involvement of neuroinflammation [56]. Whereas measuring cytokines in cerebrospinal fluid or PET scans would be useful for a more definitive assessment of neuroinflammation [53,58], these are not practical in routine clinical practice. Resting functional MRI is non-invasive and may be useful for evaluating the condition in facilities where it is available [76].

**Figure 3:**
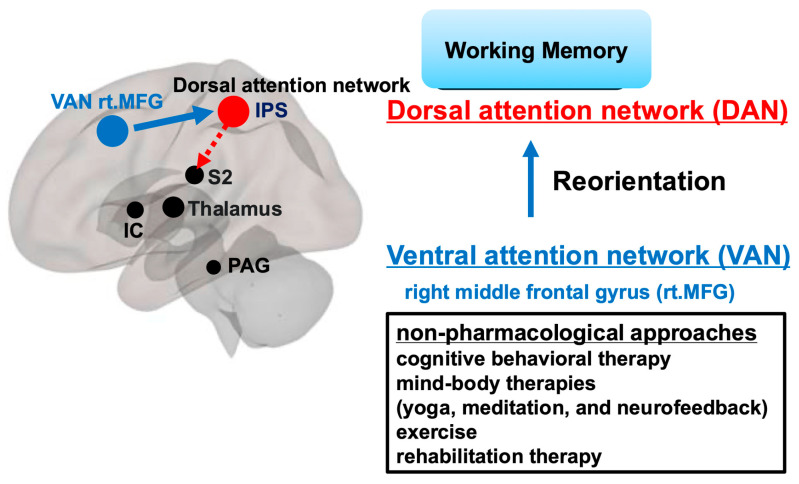
A hypothetical model for alleviating pain by reorienting pain-biased working memory. For patients whose working memory is thought to be significantly affected by pain-related attentional bias, non-pharmacological approaches aimed at reorienting working memory using the VAN-DAN interaction may be effective, such as cognitive behavioral therapy, mind–body therapies (yoga, meditation, and neurofeedback), exercise and rehabilitation therapy. This figure was created based on the paper by Aoe et al., Scientific Reports, 2024 [76] under the terms of the Creative Commons Attribution 4.0 International License. Changes were made to the original figure. IPS DAN: intraparietal sulcus in the dorsal attention network; IC: insular cortex; S2: the secondary somatosensory area; PAG: periaqueductal gray.

For patients whose working memory is thought to be significantly affected by pain-related attentional bias, non-pharmacological approaches aimed at reorienting working memory using the VAN-DAN interaction appear to be effective [98]. Cognitive behavioral therapy and mind–body therapies such as yoga, meditation, neurofeedback and expressing gratitude may be effective [102,103,104,105]. Non-pharmacological interventions, such as exercise and rehabilitation therapy, have also shown consistent benefits in reducing pain, fatigue, and improving mood, according to recent randomized trials and meta-analyses [106,107] (Figure 3). Meanwhile, in conditions such as post-viral sequelae or autoimmune mechanisms where neuroinflammation is a major driver, pharmacological therapy may be more effective. Several therapeutic approaches targeting redox imbalance have been proposed for neuroinflammation [60], including ubiquinol [108], coenzyme Q10 [109], melatonin [110], glutathione (and glutathione donors), and vitamin D [111]. However, none of these approaches has yet been clinically established. Curative treatment is currently difficult, and symptomatic treatment with analgesics remains the norm. In clinical practice and clinical trials, SNRIs such as duloxetine and milnacipran, amitriptyline, and antiepileptic drugs (e.g., pregabalin) have demonstrated efficacy in some patients, especially when tailored to individual symptom profiles, comorbidities, and psychosocial factors [104,112].

### 4.3. Limitations

This paper is not a comprehensive review that covers all research, but rather a perspective-based review that selects studies from a specific viewpoint and may therefore be subject to interpretive bias. The characteristics of FM patients may vary depending on the diagnostic criteria used in each study [113], making it difficult to evaluate the results of each study. The resting state functional MRI study discussed in this paper involved a small number of patients, and further confirmation in larger-scale studies is needed [76].

## 5. Methods

The PubMed library was searched up to 30 November 2025. The search terms were (fibromyalgia) and (nociceptive pain, neuropathic pain, or nociplastic pain). Literature relevant to the theme of this perspective was selected primarily from human studies. References cited in the selected articles were also incorporated as appropriate. The author selected studies considered representative of pain classification. Animal studies were excluded because of the difficulty of assessing model validity in relation to human FM.

## 6. Conclusions

Clinicians should consider the diverse pathophysiologies of FM and select appropriate pharmacological and non-pharmacological therapies tailored to each patient. Non-pharmacological therapies may yield more effective results if applied to patients who perceive pain intrinsically due to the influence of pain-biased working memory. For patients whose pain perception is affected by neurological disorders such as neuroinflammation, symptomatic treatments are available, but future development of disease-modifying or curative treatments is needed.

## Data Availability

No new data were created or analyzed in this study. Data sharing is not applicable to this article.

## Abbreviations

The following abbreviations are used in this manuscript:

FM	fibromyalgia
ACR	American college of rheumatology
CNS	central nervous system
TRPV1	transient receptor potential vanilloid 1
CGRP	calcitonin gene-related peptide
*5-HTTLPR*	serotonin transporter gene
*COMT*	catechol-O-methyl transferase gene
*DRD4*	dopamine receptor D4 gene
*TRPA1*	transient receptor potential ankyrin 1 gene
PET	positron emission tomography
TSPO	translocator protein
MRI	magnetic resonance imaging
DKI	diffusion kurtosis imaging
NGF	nerve growth factor
TrkA	tropomyosin receptor kinase a
PAG	periaqueductal gray
SNRIs	serotonin and noradrenaline reuptake inhibitors
HPA	hypothalamic–pituitary–adrenal axis
PL	low pain threshold
PPTs	pressure pain thresholds
PN	normal pain threshold
S2	secondary somatosensory cortex
DAN	dorsal attention network
VAN	ventral attention network
IPS	intraparietal sulcus
